# Evaluation of Low-Dose Aspirin on Pregnancy Outcomes: A Systematic Review and Meta-analysis

**DOI:** 10.34172/aim.33275

**Published:** 2025-04-01

**Authors:** Wei Wang, Guowei Chang

**Affiliations:** ^1^Department of Pharmacy, Deqing People’s Hospital, Huzhou, China

**Keywords:** Aspirin, Gestational hypertension, Preeclampsia, Pregnancy

## Abstract

**Background:**

Preeclampsia is a severe pregnancy disorder linked to high maternal and neonatal mortality. This meta-analysis evaluates the effectiveness of low-dose aspirin in reducing the occurrence of preeclampsia and associated outcomes.

**Methods:**

A total of 28 trials were included, analyzed using a random-effects model to calculate risk ratios (RR) and 95% confidence intervals (CI). The studies compared low-dose aspirin administered at or before 16 weeks of gestation to a control group. The measured parameters are the effect of low-dose aspirin on pregnancy outcomes. Study inclusion criteria consisted of studies in which low-dose aspirin was administrated at or before 16 weeks of gestation and compared to a control group.

**Results:**

Low-dose aspirin significantly reduced preterm (RR=0.52, 95% CI [0.31, 0.88]) and term preeclampsia (RR=0.97, 95% CI [0.69, 1.38]). It also decreased intrauterine growth restriction (RR=0.63, 95% CI [0.54, 0.74]). However, no significant differences were observed for postpartum hemorrhage (RR=0.71, 95% CI [0.49, 1.02]) or gestational hypertension (RR=0.65, 95% CI [0.39, 1.07]). Aspirin doses≥100 mg were more effective in reducing preterm preeclampsia risk compared to doses<100 mg, which showed variable efficacy and greater heterogeneity.

**Conclusion:**

Low-dose aspirin significantly decreases the risk of preterm and term preeclampsia but has limited impact on gestational hypertension and postpartum bleeding. Study limitations include the absence of large randomized controlled trials (RCTs) early in pregnancy (before 16 weeks) and small sample sizes in the included trials, complicating precise dose determination.

## Introduction

 Following the 20th week of pregnancy, the beginning of proteinuria and hypertension is the defining characteristic of preeclampsia, which is a multisystem disorder that occurs throughout pregnancy.^1^ Over 70 000 deaths as a result of preeclampsia occur yearly among pregnant women all over the world, and it is the most frequent form of hypertensive disease that occurs during pregnancy.There has been a correlation established between preeclampsia and increased rates of cardiovascular death in both mothers and newborns in the future.Aspirin use during pregnancy has been linked to reduced risk of preeclampsia ever since it was first reported in 1979 by Crandon and Isherwood.^2^ This association has remained in place ever since.^3^ Since that time, a number of experiments spanning several decades have been conducted to examine the possibility of low doses of aspirin preventing preeclampsia. These experiments include more than 50 individual studies and 27 meta-analyses. There is a heated debate on the effectiveness of aspirin in preventing preeclampsia and its associated outcomes. Although there have been numerous high-quality, systematic reviews multicenter randomized controlled trials (RCTs) that have included a significant number of women, the debate has not been concluded.^4,5^ The efficacy of low-dose aspirin in preventing preeclampsia may be influenced by the timing of medication initiation. This is a factor that should be considered. According to the World Health Organization (WHO), females who are at a significant risk of developing preeclampsia should take a moderate dosage of aspirin (75 mg/d) in order to prevent the condition. A recent study was conducted at multiple institutions, employing a double-blind, placebo-controlled methodology. The study included 1776 pregnant women who were carrying a single child. The investigation revealed that the utilization of aspirin can diminish the probability of acquiring early preeclampsia.^6^ From the beginning of their pregnancies until the 36th week of their pregnancies, the women were either given a placebo or a modest dose of aspirin. In spite of this, in terms of the occurrence of neonatal poor outcomes or other adverse events, there were no statistically significant differences seen between the groups.^5^ It is essential to have deeper understanding of the effects of aspirin in this context because it is currently the most effective treatment option for improving outcomes for women who are at risk of preeclampsia and the adverse sequelae associated with it. Regarding timing of therapy initiation and discontinuation, the American College of Obstetricians and Gynecologists (ACOG) and Society for Maternal-Fetal Medicine (SMFM) guidelines currently advise initiating treatment between 12 and 28 weeks of gestation, with a preference for starting low-dose aspirin before 16 weeks.^7^ Most studies, however, have focused on treatment groups recruited at or after 12 weeks, leaving the potential benefits of initiating treatment prior to 12 weeks unexamined.^8^ On the other hand, the FIGO guidelines advise discontinuing aspirin at 37 weeks or two weeks before a planned delivery to mitigate hemorrhage risk. In contrast, most trials reviewed by the United States Preventive Services Task Force (USPSTF) continued aspirin until delivery; however, eight trials stopped prophylactic aspirin earlier (some as early as 34 weeks) or when preeclampsia developed.^9^

 Regarding aspirin dose, a study by Van Doorn et al, which included preeclampsia outcomes across all gestational ages, revealed that aspirin doses below 150 mg did not effectively reduce the risk of preterm preeclampsia. Notably, only one study used a 150 mg dose, limiting the statistical power of these findings. However, this dose demonstrated a 62% reduction in preterm preeclampsia risk.^10^

 We hypothesize that the effectiveness of low-dose aspirin in reducing the incidence of preeclampsia may vary depending on the specific dosage administered and the timing of initiation, particularly when started at or before 16 weeks of gestation.

 The purpose of this meta-analysis was to estimate the efficacy of low doses of aspirin in preventing preeclampsia, as well as its related adverse events for both the mother and the newborn. This was accomplished by conducting an analysis of a large number of original studies, including newly published studies that had not been discussed in any of the previous meta-analyses concerning aspirin therapy prior to four months of gestation.

## Materials and Methods

###  Study Design

 This meta-analysis is conducted in accordance with epidemiological guidelines and follows a study methodology that was established beforehand. Several databases, including OVID, the Cochrane Library, PubMed, Embase, and Google Scholar, were consulted in order to obtain the necessary information.^11,12^

###  Data Pooling

**Figure 1:**
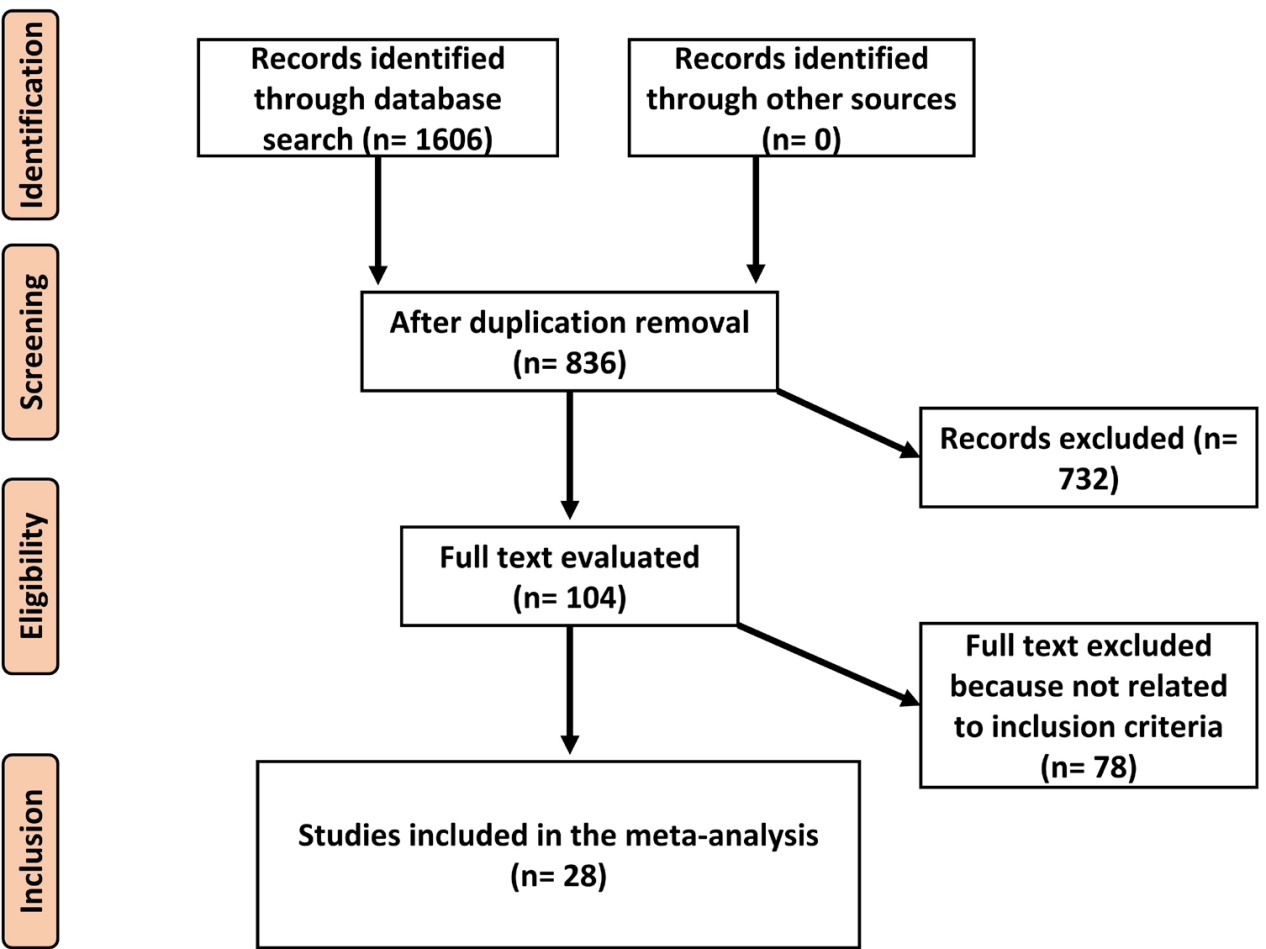
Schematic Diagram of the Study Procedure

 The study includes clinical trials that examined the impact of low-dose aspirin, which was started at 16 weeks of pregnancy or earlier, on the occurrence of preeclampsia (both term and preterm), gestational hypertension, and postpartum hemorrhage. Only human studies without language restrictions were considered. Reviews, editorials, and letters to editors were excluded. The comprehensive methodological approach is illustrated in Figure 1.

###  Eligibility and Inclusion Criteria

 This summary evaluates the effect of low doses of aspirin on pregnancy consequences related to preeclampsia and gestational complications. Sensitivity analysis included only studies comparing aspirin to placebo in terms of preterm and term preeclampsia, restriction of intrauterine growth, and gestational complications. The inclusion criteria for the meta-analysis were: retrospective, prospective, or cohort studies; pregnant participants receiving low-dose aspirin starting at 16 weeks of gestation or earlier; and comparison of preeclampsia incidence in preterm and term definitions with placebo regarding gestation and neonatal complications. The exclusion criteria were: studies assessing high-dose aspirin or starting after 16 weeks of gestation; reviews, letters, books, and book chapters. In addition, grey literature and unpublished studies were excluded from this review. Excluding grey literature was a deliberate choice to maintain the quality and consistency of evidence as grey literature often lacks the rigorous peer-review process required by academic journals. In addition, grey literature can be difficult to obtain, verify, or fully assess due to limited distribution and incomplete data.

###  Identification

 A search protocol based on the PICOS framework was developed: P (population): pregnant subjects; I (intervention/exposure): low-dose aspirin; C (comparison): risk of preeclampsia, intrauterine growth restriction (IUGR), and gestational complications; S (study design): prospective studies. A comprehensive search of databases (OVID, PubMed, Cochrane Library, Embase, and Google Scholar) was conducted through April 2024 using specified keywords. Studies were screened, and relevant articles were included in a reference management program. Two authors independently reviewed and identified eligible studies.

 The search strategy employed Boolean operators to combine key terms, including ‘aspirin’, ‘preeclampsia’, ‘postpartum hemorrhage’, ‘pregnancy’, and ‘intrauterine growth’. This approach was designed to maximize the retrieval of relevant studies, ensuring a comprehensive and systematic inclusion of literature pertinent to the review’s objectives. Terms were combined using Boolean logic (e.g. “AND,” “OR”) to refine the search and capture a wide range of studies related to aspirin’s effects on maternal and fetal health outcomes (Table 1).

**Table 1:** Databases Search Strategy

**Database**	**Search Strategy**
PubMed	#1 "Aspirin"[MeSH Terms] OR/AND "preeclampsia"[All Fields] #2 "postpartum hemorrhage"[MeSH Terms] OR "pregnancy"[All Fields] #3 "intrauterine growth"[MeSH Terms] OR/AND "Aspirin"[All Fields] #4 #1, #2, AND #3
OVID	#1 "Aspirin"[MeSH Terms] OR/AND "preeclampsia"[All Fields] #2 "postpartum hemorrhage"[MeSH Terms] OR "pregnancy"[All Fields] #3 "intrauterine growth"[MeSH Terms] OR/AND "Aspirin"[All Fields] #4 #1, #2, AND #3
Google Scholar	#1 " Aspirin " OR " preeclampsia " #2 " postpartum hemorrhage " OR " pregnancy " OR" intrauterine growth" #3 #1 AND #2
Embase	' Aspirin /exp OR preeclampsia’ #2 '' postpartum hemorrhage '/exp OR ' pregnancy '/exp OR ' intrauterine growth" #3 #1 AND #2
Cochrane library	(Aspirin):ti,ab,kw (preeclampsia): ti,ab,kw (Word variations have been searched) #2 (' postpartum hemorrhage):ti,ab,kw OR (pregnancy): ti,ab,kw (Word variations have been searched) #3 #1 AND #2

ti,ab,kw: terms in either title or abstract or keyword fields, exp: exploded indexing term.

###  Screening

 The procedure of extracting data entailed gathering information about the study and the characteristics of the subjects in a format that was standardized. This included the surname of the first author, the length of time the study was conducted, the year it was published, the nation in which the research was carried out, the type of population, the total number of subjects, the qualitative and quantitative evaluation methods that were utilized, the design of the study, the demographic data, the clinical and treatment characteristics, the source of information, the outcomes that were measured, and the methodological quality that was evaluated by two authors.

###  Statistical Analysis

 The findings from the trials included in the analysis were thoroughly examined using random model of analysis, and the relative risk (RR) and its 95% confidence interval (CI) were calculated. RR was the main statistical measure utilized to evaluate the study findings. In order to achieve a thorough and accurate statistical analysis and draw reliable scientific results, the research was conducted using a random-effects model. The constrained maximum-likelihood estimator was used to measure the level of variability among studies. To evaluate the variability among the trials included, the I^2^ statistic was performed. If heterogeneity was found (tau^2^ > 0), a prediction interval for the genuine effect sizes was also computed. The importance and reliability of individual investigations and their outcomes were further assessed by employing studentized residuals and Cook’s distances.

 Sensitivity analysis was conducted to evaluate the robustness of the findings and to understand how variations in study design and aspirin dosage influenced the outcomes. This analysis involved subgroup stratification based on aspirin dosage, comparing the effects of doses ≥ 100 mg to those < 100 mg. Additionally, the analysis was further stratified by specific pregnancy outcomes, including preterm preeclampsia, term preeclampsia, IUGR, postpartum hemorrhage, and gestational hypertension, to provide a comprehensive understanding of the variations in the results.

 A subgroup analysis was conducted to evaluate the differential effects of low-dose aspirin on specific pregnancy outcomes based on dosage categories. Subgroups were stratified into doses of aspirin ≥ 100 mg and < 100 mg, as well as outcomes for preterm and term preeclampsia, and IUGR. This analysis aimed to provide a nuanced understanding of the variations in outcomes attributable to different aspirin dosages and their timing during pregnancy.

 The statistical analyses were conducted using a software (Jamovi), which ensured rigorous and uniform analytical processes.

## Results

###  Study Selection and Inclusion

 Following a rigorous screening and identification process, a total of 28 studies were included in the current meta-analysis (Table 2).^5,13-39^

**Table 2:** Characteristics of Included Studies

**Study**	**Year**	**Aspirin group**	**Control group**	**Total**	**Aspirin dose**	**Pre-Risk inclusion criteria**	**Aspirin initiation time (wk)**
Rolnik et al^5^	2017	798	822	1620	≥ 100	High-risk pregnant subjects (chronic HTN, gestational HTN, cardiovascular diseases, endocrine disease, or fetal growth restriction)	11-14 weeks
Huai et al^13^	2020	95	95	190	60	High-risk pregnant subjects (chronic HTN, gestational HTN, cardiovascular diseases, endocrine disease, or fetal growth restriction)	16 weeks or less
Gu et al^14^	2020	821	284	1105	25, 50, and 75 mg	High-risk pregnant subjects (chronic HTN, gestational HTN, cardiovascular diseases, endocrine disease, or fetal growth restriction)	12 weeks
Abdi et al^15^	2020	43	43	86	80	High-risk pregnant subjects (chronic HTN, gestational HTN, cardiovascular diseases, endocrine disease, or fetal growth restriction)	12-15 weeks
Stanescu et al^16^	2015	100	50	150	≥ 100	High-risk pregnant subjects (chronic HTN, gestational HTN, cardiovascular diseases, endocrine disease, or fetal growth restriction)	12 weeks
Scazzocchio et al^17^	2017	80	75	155	≥ 100	Abnormal Doppler in the first trimester. High-risk pregnant subjects (chronic HTN, gestational HTN, cardiovascular diseases, endocrine disease, or fetal growth restriction)	11-14 weeks
Odibo et al^18^	2015	16	14	30	80	High-risk pregnant subjects (chronic HTN, gestational HTN, cardiovascular diseases, endocrine disease, or fetal growth restriction)	11-13 weeks
Villa et al^19^	2013	61	60	121	≥ 100	Abnormal uterine artery Doppler. High-risk pregnant subjects (chronic HTN, gestational HTN, cardiovascular diseases, endocrine disease, or fetal growth restriction)	12-13 weeks
Ayala et al^20^	2013	176	174	350	≥ 100	High-risk pregnant subjects (chronic HTN, gestational HTN, cardiovascular diseases, endocrine disease, or fetal growth restriction)	16 weeks or less
Zhao et al^21^	2012	118	119	237	75	High-risk pregnant subjects (chronic HTN, gestational HTN, cardiovascular diseases, endocrine disease, or fetal growth restriction)	13-16 weeks
Jamal et al^22^	2012	35	35	70	80	Polycystic ovary syndrome	6-12 weeks
Mesdaghinia et al^23^	2011	40	40	80	80	Abnormal uterine artery Doppler	12-16 weeks
Bakhti et al^24^	2011	82	82	164	≥ 100	High-risk pregnant subjects (chronic HTN, gestational HTN, cardiovascular diseases, endocrine disease, or fetal growth restriction)	8-10 weeks
Ebrashy et al^25^	2005	73	63	136	75	Abnormal uterine artery Doppler. High-risk pregnant subjects (chronic HTN, gestational HTN, cardiovascular diseases, endocrine disease, or fetal growth restriction)	14-16 weeks
Chiaffarino et al^26^	2004	16	19	35	≥ 100	High-risk pregnant subjects (chronic HTN, gestational HTN, cardiovascular diseases, endocrine disease, or fetal growth restriction)	Less 14 weeks
Vainio et al^27^	2002	43	43	86	0.5mg/kg/day	High-risk pregnant subjects (chronic HTN, gestational HTN, cardiovascular diseases, endocrine disease, or fetal growth restriction)	12-14 weeks
Golding et al^28^	1998	1009	988	1997	60	Nulliparity	12-16 weeks
Dasari et al^29^	1998	25	25	50	≥ 100	Nulliparity	12 weeks
Caritis et al^30^	1998	265	258	523	60	High-risk pregnant subjects (chronic HTN, gestational HTN, cardiovascular diseases, endocrine disease, or fetal growth restriction)	13-16 weeks
Tulppala et al^31^	1997	33	33	66	50	Previous consecutive miscarriage	Less than 7 weeks
Hermida et al^32^	1997	50	50	100	≥ 100	High-risk pregnant subjects (chronic HTN, gestational HTN, cardiovascular diseases, endocrine disease, or fetal growth restriction)	12-16 weeks
Augus et al^33^	1994	24	25	49	≥ 100	High-risk pregnant subjects (chronic HTN, gestational HTN, cardiovascular diseases, endocrine disease, or fetal growth restriction)	13-15 weeks
Siba et al^34^	1993	320	324	644	60	Nulliparity	13-16 weeks
porreco et al^35^	1993	48	42	90	80	Nulliparity multiple gestation	Less 16 weeks
Michael et al^36^	1992	55	55	110	≥ 100	High-risk pregnant subjects (chronic HTN, gestational HTN, cardiovascular diseases, endocrine disease, or fetal growth restriction)	Less than 16 weeks
Azar et al^37^	1990	46	45	91	≥ 100	High-risk pregnant subjects (chronic HTN, gestational HTN, cardiovascular diseases, endocrine disease, or fetal growth restriction)	16 weeks
Benigni et al^38^	1989	17	16	33	60	High-risk pregnant subjects (chronic HTN, gestational HTN, cardiovascular diseases, endocrine disease, or fetal growth restriction)	12 weeks
Beaufils et al^39^	1985	48	45	93	≥ 100	High-risk pregnant subjects (chronic HTN, gestational HTN, cardiovascular diseases, endocrine disease, or fetal growth restriction)	14 weeks

HTN: Hypertension.

###  Risk of Preeclampsia

**Figure 2:**
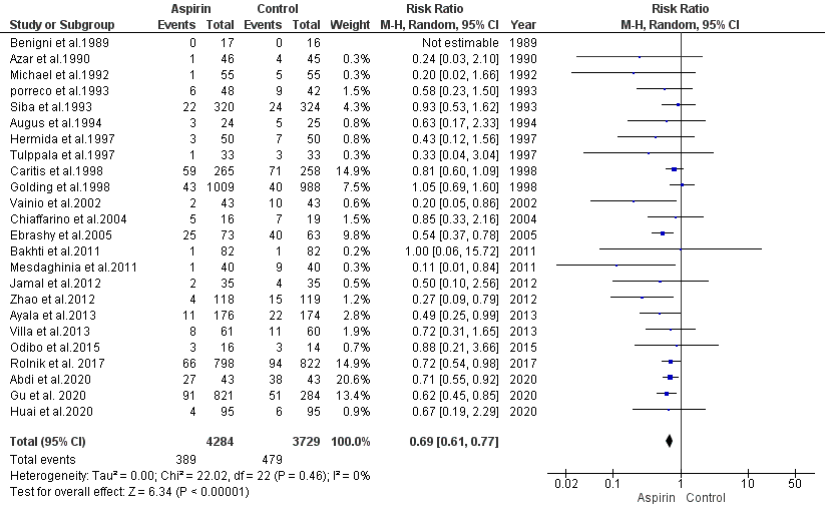
Impact of Low-Dose Aspirin on the Risk of Preeclampsia

 Our analysis used a random-effects model to determine the association between aspirin usage and the risk of preeclampsia. This analysis synthesized data from 24 studies. The findings indicated a significant decline in the risk of preeclampsia among individuals who received aspirin in comparison with the control group (RR = 0.69, 95% CI: 0.61, 0.77), as illustrated in Figure 2. The regression test identified funnel plot asymmetry (*P*= 0.01), suggesting potential publication bias, while the rank correlation test did not indicate such a bias (*P*= 0.42).

###  Risk of Preterm Preeclampsia

**Figure 3:**
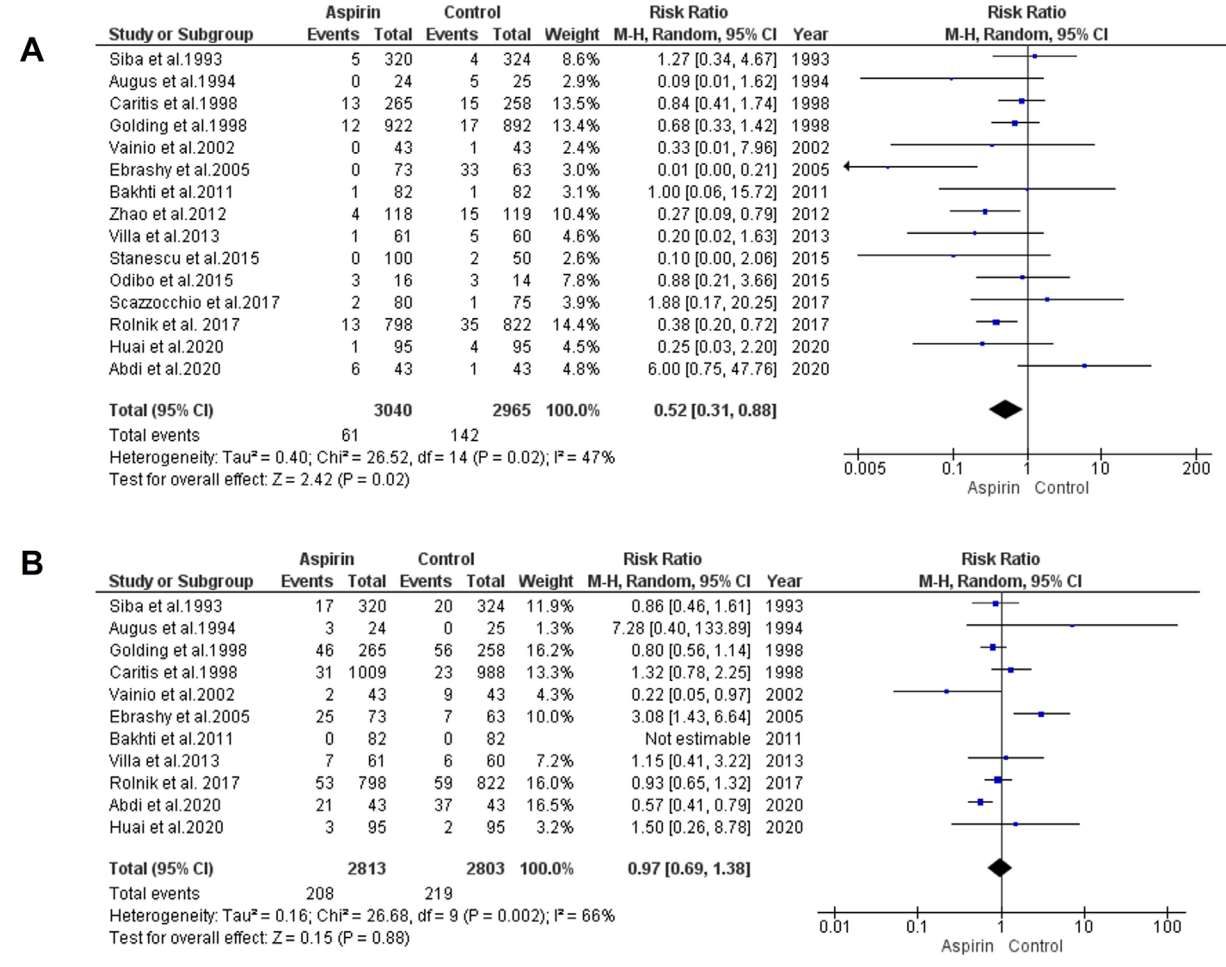
Impact of Low-Dose Aspirin on the Risk of Preterm (a) and Term Preeclampsia (b)

 The impact of low-dose aspirin on the risk of preterm preeclampsia was considered by combining data from 15 studies using a model with random-effects. The results demonstrated a significant decline in the risk of preterm preeclampsia in the aspirin group compared to the control group (RR = 0.52, 95% CI: 0.31, 0.88), as depicted in Figure 3a. Both the rank correlation and regression tests showed non-significant results for publication bias (*P*= 0.44 and *P*= 0.34, respectively).

###  Risk of Term Preeclampsia

 To estimate the effect of low-dose aspirin on the risk of term preeclampsia, data from 11 trials were analyzed using a model with random-effects. The results indicated no significant decline in the risk of term preeclampsia (RR = 0.97, 95% CI: 0.69, 1.38), as presented in Figure 3b. Both the rank correlation and regression tests revealed no significant evidence of publication bias (*P*= 0.65 and*P*= 0.71, respectively).

###  Risk of Intrauterine Growth Restriction

**Figure 4:**
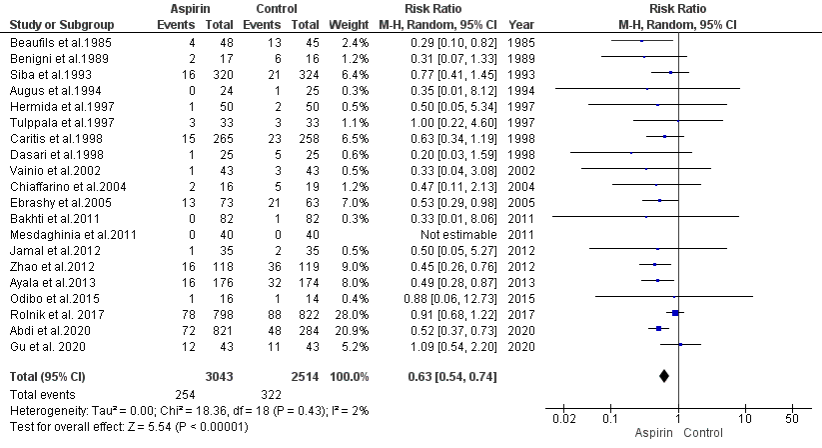
Impact of Low-dose Aspirin on the Risk of Intrauterine Growth Restrictions

 The link between aspirin usage and the restriction of intrauterine growth risk was examined by pooling data from 21 trials using a random-effects model. The analysis revealed a significant reduction in the risk of IUGR among those who used aspirin compared to the control group (RR = 0.63, 95% CI: 0.54, 0.74), as illustrated in Figure 4. The rank correlation and regression tests both indicated non-significant results for publication bias (*P*= 0.41 and *P*= 0.29, respectively).

###  Risk of Gestational Hypertension

**Figure 5:**
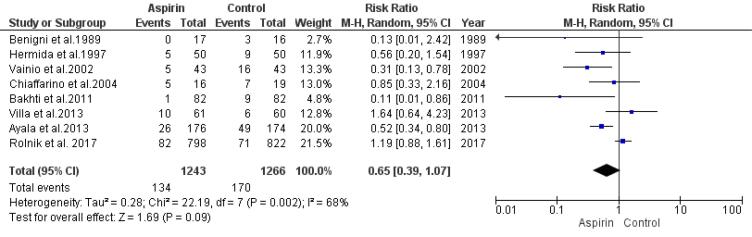
Impact of Low-dose Aspirin on the Risk of Gestational Hypertension

 The effect of low doses of aspirin on the risk of developing gestational hypertension was assessed by analyzing data from eight studies using a random-effects model. The findings showed no significant decline in the risk of gestational hypertension in the aspirin group compared to the control group (RR = 0.65, 95% CI: 0.39, 1.07), as depicted in Figure 5. There was no significant indication of publication bias, as evidenced by the rank correlation and regression tests (*P*= 0.72 and *P*= 0.94, respectively).

###  Risk of Postpartum Hemorrhage

**Figure 6:**
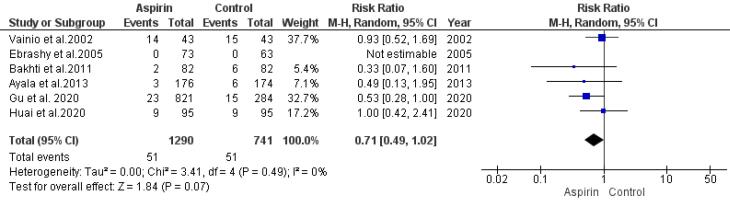
Impact of Low-dose Aspirin on the Risk of Postpartum Hemorrhage

 An analysis model was employed to evaluate the association between aspirin usage and the risk of postpartum hemorrhage. Data from six studies were synthesized for this analysis. The results indicated a statistically insignificant decline in the risk of postpartum hemorrhage (RR = 0.71, 95% CI: 0.49, 1.02), as illustrated in Figure 6. Both the rank correlation and regression tests did not indicate significant publication bias (*P*= 0.99 and *P*= 0.91, respectively).

 The subgroup analysis to evaluate the association between aspirin dosage and the risk of preterm preeclampsia, term eclampsia, and IUGR showed the following findings: for preterm preeclampsia, aspirin doses ≥ 100 mg significantly reduced the risk, (RR = 0.77, 95% CI [0.66, 0.87], *P* < 0.0001), and no significant heterogeneity was observed (I^2^ = 16.76%). In contrast, doses < 100 mg demonstrated a non-significant risk reduction (RR = 0.89, 95% CI [0.61, 1.24], *P* = 0.27), accompanied by high heterogeneity (I^2^ = 98.90%). Regarding term eclampsia, neither dose showed a significant effect, (RR = 1.00, 95% CI [0.74, 1.34], *P* = 0.95 for doses ≥ 100 mg and RR = 0.96, 95% CI [0.85, 1.08], *P* = 0.52 for doses < 100 mg). For IUGR, aspirin doses ≥ 100 mg significantly reduced the risk (RR = 0.74, 95% CI [0.61, 0.85], *P* = 0.005), while doses < 100 mg also showed significant risk reduction (RR = 0.76, 95% CI [0.63, 0.91], *P* = 0.003). No significant publication bias was identified for any subgroup based on rank correlation or regression tests

###  Quality and Risk of Bias Assessment

**Figure 7:**
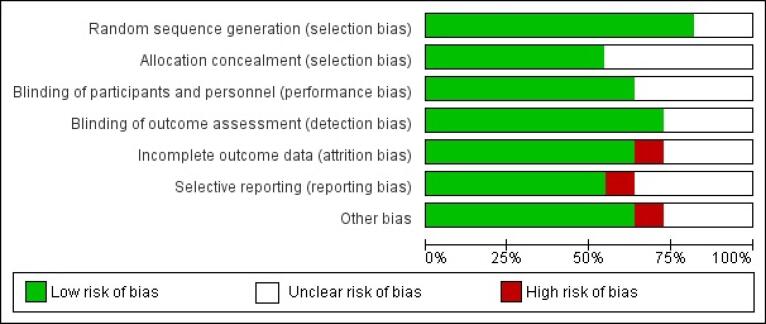
Quality of Recruited Studies

 The quality assessment of the recruited and analyzed studies and the evaluation of the risk of bias demonstrated high-quality studies with a substantial percentage of studies exhibiting a low risk of bias across all domains, as illustrated in Figure 7.

###  Publication Bias

 This was further supported by a significant *P* value in Egger’s test (*P* = 0.01), while Begg’s test did not indicate bias (*P* = 0.42). For preterm preeclampsia, no significant publication bias was detected (Egger’s test, *P* = 0.34; Begg’s test, *P* = 0.44). Similarly, no significant publication bias was observed for term preeclampsia, IUGR, gestational hypertension, and postpartum hemorrhage, as indicated by non-significant *P *values in both Egger’s and Begg’s tests.

 The comprehensive analysis provides robust evidence supporting the beneficial effects of aspirin in lowering the risk of preeclampsia and IUGR. However, the non-significant findings for term preeclampsia, gestational hypertension, and postpartum hemorrhage warrant further investigation with larger sample sizes and well-designed studies to confirm these results.

## Discussion

 The current investigation included a total of 28 clinical investigations, which resulted in the recruitment of a total of 8156 participants from a variety of nations. When aspirin was administered at low dosages, it was found to be associated with a significantly (*P* < 0.001) reduced risk of preeclampsia, as well as term and preterm preeclampsia, and IUGR. Although there was no evident difference between the interventional group and the control group in terms of postpartum hemorrhage and gestational hypertension, the interventional group was found to have a higher incidence of both of these conditions. The data strongly support the concept that early administration of low-dose aspirin enhances placental formation and development.

 The findings of subgroup analysis highlight a dose-dependent effect of aspirin on preeclampsia and IUGR outcomes. Aspirin doses ≥ 100 mg demonstrated statistically significant reductions in risk for preterm preeclampsia and IUGR, with low heterogeneity and consistent results across studies. These results underscore the potential benefits of higher aspirin doses in preventing these complications, likely due to enhanced anti-inflammatory and antiplatelet effects. Conversely, doses < 100 mg showed variable outcomes with substantial heterogeneity, especially for preterm preeclampsia, term eclampsia, and IUGR. The presence of outliers and influential studies in this subgroup may have contributed to the variability, suggesting that the lower dose may not be sufficient for uniform efficacy across diverse populations. The findings’ generalizability to broader populations remains uncertain due to the limited number of large-scale RCTs. This lack of RCTs contributes to some uncertainty, particularly regarding nonsignificant results.

 For term eclampsia, no significant differences were observed regardless of aspirin dosage, indicating a potential limitation of aspirin in preventing term-related hypertensive complications. The primary outcomes of the study demonstrated a significant reduction in the incidence of preeclampsia among participants who received aspirin compared to the control group. This finding aligns with existing evidence suggesting the efficacy of low-dose aspirin in mitigating the risk of preeclampsia, particularly in high-risk populations. The magnitude of the effect underscores the importance of early intervention and adherence to prophylactic regimens for those identified at risk through clinical screening criteria.

 In contrast, the secondary outcomes, including gestational hypertension and postpartum hemorrhage, did not show statistically significant changes between the aspirin and control groups. This lack of significant effect suggests that the impact of aspirin may more specifically target the pathophysiological pathways associated with preeclampsia, rather than general hypertensive disorders or hemorrhagic complications of pregnancy.^40,41^

 The absence of significant changes in gestational hypertension may reflect differences in the underlying mechanisms of these conditions.^41^ Preeclampsia is characterized by endothelial dysfunction and placental pathology, processes that aspirin may more effectively influence through its anti-inflammatory and antiplatelet actions.^42,43^ In contrast, gestational hypertension, which lacks proteinuria or severe systemic manifestations, may not benefit from aspirin in the same way.

 Similarly, lack of an effect on postpartum hemorrhage could indicate that aspirin does not exacerbate bleeding risks in the obstetric population at the administered dosage, which is reassuring in terms of clinical safety.^44^ It also suggests that the prophylactic benefits of aspirin are confined to its role in addressing preeclampsia-specific mechanisms without adverse trade-offs in terms of hemorrhagic outcomes. It is crucial for women who are susceptible to preeclampsia to be cognizant of this knowledge in order to make well-informed choices about their prenatal care prior to delivery. However, there is considerable debate on the efficacy of low-dose aspirin in preventing preeclampsia and the associated risks for both women and newborns. In order to provide additional evidence that aspirin is effective for this indication, additional clinical trials of a higher quality and a larger sample size are required. In recent years, antiplatelet medications have been the subject of intense research because of their potential to prevent or delay the onset of preeclampsia and the complications that it can cause. Despite the fact that a number of research have discovered significant benefits,^45-47^ other investigations have not found any.^48-50^ A prior meta-analysis of individual patient data revealed a moderate yet consistent decrease in the RR for adverse outcomes in mothers and neonates. The present study assessed the effectiveness of low-dose aspirin treatment in preventing preeclampsia in pregnant patients who were at risk of developing preeclampsia and whose preeclampsia began at 16 weeks of pregnancy. The findings of the meta-analysis were consistent with those observed in the prior meta-analysis.^51^ At a recent multicenter, double-blind, placebo-controlled study, which included 1776 women with singleton pregnancies who were at high risk for preterm preeclampsia, it was discovered that, in comparison to the placebo, low-dose aspirin significantly reduced the number of instances in which this diagnosis was made.^5^ Because it satisfied the inclusion criteria, this most recent study was incorporated into the analysis that is being presented here. Previous research has demonstrated that beginning to take aspirin at approximately 16 weeks of pregnancy can assist in lowering the chance of developing preeclampsia, and the findings of this study offer further evidence to support those findings. A number of abnormalities, such as angiogenesis, oxidative stress, and inflammation, are suggested to play a part in the development of preeclampsia, despite the fact that the exact origin of the condition has not yet been identified. Among the numerous supplements and medications that have been attempted for the primary and secondary prevention of preeclampsia,^52^ antihypertensives, calcium,^53^ and the antioxidant vitamins C and E^54^ are just a few examples. However, none of these treatments or supplements have proved successful. During the first stages of the inquiry, pravastatin had favorable results.^55^ However, before it can be integrated into standard clinical practice, its benefits (and safety in pregnancy) need to be explored in a large and well-designed RCT with a sample size that is large enough to obtain high statistical power. Having a healthy pregnancy is contingent upon the implantation and placentation processes occurring in a normal manner. By the tenth week of pregnancy, it is anticipated that the initial wave of trophoblast invasion will have been completed, and it will continue until the week after.^55,56^ Both the endothelial function and the early development of the placenta have been found to be improved by the use of aspirin.^57,58^ It was established that the risk of preeclampsia and other adverse outcomes for both the mother and the infant was dramatically reduced when low-dose aspirin was started before 16 weeks of gestation. However, this was not the case when it was administered after 16 weeks of gestation, as stated by Bujold et al who conducted the study.^59^ On the other hand, a recent meta-analysis discovered that taking low-dose aspirin had the same effect on preventing preeclampsia and the repercussions that are connected with it, regardless of whether the medication was started before or after 16 weeks with the pregnancy.^60^ Due to the fact that the latter review only included individuals who had previously been treated with antiplatelet medications (such as dipyridamole or low molecular weight heparin), it is likely that these contradictory findings can be explained accordingly. In addition, studies that did not restrict the beginning of antiplatelet medication to the first sixteen weeks of pregnancy were included in the meta-analysis of individual participant data that was conducted later. The current study indicated that low-dose aspirin reduced the risk of these issues, which is consistent with the findings of a previous meta-analysis that found that antiplatelet drugs reduced the risk of preterm delivery, small for gestational age (SGA), and other negative outcomes for both the mother and the fetus.^51^ The fact that this meta-analysis only evaluated a small number of studies was one of its most significant shortcomings. There appears to be some diversity among the studies that were included, particularly with regard to the negative outcomes that were experienced by mothers and their newborn children.

 A significant drawback of this meta-analysis is the absence of major RCTs that recruited individuals at an early stage of pregnancy (within 16 weeks of gestation). The majority of the evidence comes from RCTs that are either small to moderate in size or subsets of patients in larger trials. The inability to determine the appropriate dosage of aspirin is due to the small sample numbers and the inexplicable heterogeneity that exists within some subgroups.

## Conclusion

 Aspirin taken in low doses throughout pregnancy has been shown to dramatically lower the chance of developing preterm and term preeclampsia. It does not have a substantial impact on the complications of postpartum hemorrhage or gestational hypertension. In order to validate these findings, more research involving other centers is required. Aspirin at doses ≥ 100 mg is associated with significant risk reduction for preterm preeclampsia and IUGR, supporting its preferential use in high-risk populations, while lower doses exhibit inconsistent effects and should be used cautiously. While low-dose aspirin shows promise in reducing preeclampsia risk, the limitations of the current evidence, including small study sizes and methodological inconsistencies, necessitate cautious interpretation and suggest that additional large RCTs are warranted
